# Heart-Type Fatty Acid–Binding Protein as a Marker of Subclinical Cardiac Dysfunction and Cardiorenal Interaction in Autosomal Dominant Polycystic Kidney Disease

**DOI:** 10.3390/life16060966

**Published:** 2026-06-08

**Authors:** Bogdan D. Agavriloaei, Stefan Iliescu, Gianina Dodi, Claudia M. A. Zaharie, Luminita E. Voroneanu, Mugurel Apetrii, Călin Namolovan, Andreea S. Covic, Cornel Moroșanu, Mehmet Kanbay, Adrian C. Covic

**Affiliations:** 1 Grigore T. Popa University of Medicine and Pharmacy Iasi, 700115 Iasi, Romania; bogdan-dumitru.agavriloaei@umfiasi.ro (B.D.A.); stefan.iliescu@umfiasi.ro (S.I.); elena.voroneanu@umfiasi.ro (L.E.V.); mugurel.apetrii@umfiasi.ro (M.A.); calin.namolovan@email.umfiasi.ro (C.N.); andreea-simona.covic@umfiasi.ro (A.S.C.); 2 Dr. C.I. Parhon Clinical Hospital, 700503 Iasi, Romania; zaharieclaudia@yahoo.com (C.M.A.Z.); corneliumorosanu@gmail.com (C.M.); 3 School of Medicine, Koç University, 34450 Istanbul, Turkey; mkanbay@ku.edu.tr

**Keywords:** autosomal dominant polycystic kidney disease, cardiovascular disease, H-FABP, cardiovascular risk

## Abstract

(1) Background: Cardiovascular disease represents the leading cause of morbidity and mortality in patients with autosomal dominant polycystic kidney disease (ADPKD), often developing early in the disease course, even in the presence of preserved renal function. We aimed to evaluate circulating heart-type fatty acid–binding protein (H-FABP) as a marker of subclinical cardiac involvement and cardiorenal interaction in ADPKD. (2) Methods: In this single-center observational study, 80 adult patients with ADPKD receiving tolvaptan therapy were evaluated using echocardiography, renal function parameters, and circulating H-FABP levels. Associations between H-FABP and echocardiographic indices of cardiac structure and function, as well as renal parameters, were assessed using linear regression models. In addition, a composite severity score integrating CKD stage and H-FABP levels was constructed to assess the combined cardiorenal burden. (3) Results: Higher H-FABP concentrations were significantly associated with echocardiographic markers suggestive of subclinical cardiac involvement, particularly parameters related to impaired myocardial relaxation, including lower E/A ratio and reduced tissue Doppler e′ velocities. These associations remained significant after adjustment for renal function and relevant clinical covariates. In parallel, H-FABP levels were also associated with markers of renal disease severity, including lower baseline eGFR and greater total kidney volume. The composite severity score showed a graded association with echocardiographic parameters, with a progressive trend toward less favorable diastolic indices as risk categories increased. These findings suggest a potential complementary role for H-FABP in the integrated evaluation of cardiorenal involvement in ADPKD. Given the cross-sectional design and single-centre setting, these results should be considered hypothesis-generating and require prospective validation in larger, independent cohorts.

## 1. Introduction

Autosomal dominant polycystic kidney disease (ADPKD) represents the most frequent inherited cause of end-stage kidney disease worldwide [1]. It is a progressive, multisystem disorder characterized by renal cyst development and kidney enlargement, often accompanied by extrarenal organ involvement, including the cardiovascular system, with a reported prevalence among diagnosed individuals ranging from approximately 1 in 400 to 1 in 1000 across all populations [2].

The cardiac manifestations, such as, hypertension, which may lead to left ventricular hypertrophy and diastolic dysfunction, valvular abnormalities, aortic aneurysm, and pericardial effusion, result in mortality and morbidity in ADPKD patients [3].

Hypertension is highly predominant in ADPKD and frequently develops early in the course of the disease, preceding any significant decline in renal function [4]. It has been reported to affect approximately 10–20% of children and more than 60% of adults with preserved glomerular filtration rate [5]. In parallel, patients with ADPKD exhibit an increased left ventricular mass index and a high prevalence of left ventricular hypertrophy, reflecting early cardiac remodeling that is closely associated with the high burden of hypertension in this population [6].

Although ADPKD is a multisystem disorder, patients who progress to end-stage renal disease exhibit a significantly lower overall mortality on renal replacement therapy compared with non-diabetic ESRD populations, with mortality being driven predominantly by cardiovascular and cerebrovascular causes rather than by extrarenal cystic complications [7]. Cardiovascular involvement, therefore, represents a central component of disease burden in ADPKD, developing early in the disease course and constituting a major risk factor, even among patients with preserved renal function. This elevated cardiovascular risk represents a subject of increasing interest not only for scientific purposes but also for physicians to prioritize early screening and timely management to improve patient’s outcomes [8].

In ADPKD, imaging-based markers have been widely used to characterize both renal and cardiovascular involvement, with echocardiographic parameters allowing the detection of structural and functional cardiac alterations; however, these measures primarily reflect established remodelling and are limited in their ability to identify early or subclinical myocardial injury [9].

Fatty acid–binding proteins (FABPs) are a family of small cytosolic proteins that were first identified in 1972 and are involved in the intracellular transport, storage, and metabolism of long-chain fatty acids and other lipophilic molecules. They are expressed in a tissue-specific manner and play an important role in cellular energy homeostasis, signal transduction, and the regulation of inflammatory and metabolic pathways [10].

Heart-type fatty acid–binding protein (H-FABP) is predominantly expressed in cardiomyocytes, where it facilitates fatty acid uptake and intracellular trafficking, reflecting the high dependence of myocardial tissue on fatty acid oxidation for energy production [11]. Owing to its low molecular weight and cytosolic localization, H-FABP is rapidly released into the circulation following myocardial injury, including subtle or subclinical forms of cardiomyocyte damage [12].

The elimination of H-FABP occurs predominantly via renal filtration, given its low molecular weight (~15 kDa), which allows free passage through the glomerular membrane [13]. Consequently, circulating H-FABP levels are expected to rise progressively as renal function declines. Supporting this, Yu et al. demonstrated that plasma FABP-3 concentrations were independently and significantly associated with eGFR stage in patients with type 2 diabetes mellitus, with levels increasing across CKD stages G2 through G4 [14]. In a large multicenter prospective cohort of patients with chronic coronary syndrome, Yeh et al. further showed that higher serum FABP-3 independently predicted a clinically meaningful decline in eGFR over 36 months of follow-up [15]. Moreover, Shirakabe et al. demonstrated that H-FABP levels, unlike BNP or troponins, specifically predicted true worsening of renal function in patients hospitalized for acute heart failure, suggesting that H-FABP may capture a distinct cardiorenal signal beyond passive retention [16]. Beyond its established role as an early biomarker of acute myocardial injury, circulating H-FABP levels have been shown to reflect chronic myocardial stress and structural cardiac alterations, while also being influenced by renal function due to partial renal clearance. Collectively, these characteristics position H-FABP as a potential integrative biomarker at the intersection of cardiac and renal pathology, particularly in conditions characterized by early cardiovascular involvement and progressive kidney disease, such as ADPKD [3].

In a prospective cohort of patients undergoing cardiac surgery, higher preoperative serum H-FABP concentrations were associated with an increased incidence of postoperative acute kidney injury and longer intensive care unit stay, supporting a potential role for H-FABP as a predictive biomarker of AKI in this setting [17]. To date, there are no studies specifically evaluating H-FABP in patients with ADPKD, leaving its potential role in this population largely unexplored.

Therefore, the present study aimed to comprehensively evaluate circulating H-FABP levels in a well-characterized cohort of 80 patients with ADPKD undergoing treatment with tolvaptan, and to examine their associations with renal function and echocardiographic parameters. By doing so, we sought to explore the potential role of H-FABP as an integrative biomarker of cardiorenal involvement in ADPKD, reflecting both subclinical myocardial stress and the underlying renal disease burden.

## 2. Materials and Methods

### 2.1. Study Design and Population

We conducted a single-center, observational cohort study in a clinic-based population of adults with ADPKD receiving tolvaptan therapy, evaluated either during hospitalization or at scheduled ambulatory visits at the Dr. C.I. Parhon Clinical Hospital in Iasi, Romania. The initial study population comprised of 120 patients with ADPKD receiving tolvaptan. The primary analysis was restricted to 80 patients who fulfilled all predefined inclusion and exclusion criteria and had contemporaneous measurements of the variables of interest, including circulating H-FABP, renal function parameters, and echocardiographic assessments, thereby constituting the final analytic cohort.

Inclusion criteria

Patients were eligible for inclusion if they met all the following criteria:

• adults (≥18 years) with a confirmed diagnosis of ADPKD on imaging criteria and/or genetic testing;
• treatment with tolvaptan, with receipt of at least one dose at the time of enrolment;
• availability of circulating H-FABP measurement obtained according to the study protocol;
• concurrent assessment of renal function, including serum creatinine and estimated glomerular filtration rate (eGFR) calculated using the CKD-EPI 2021 equation [18];
• availability of transthoracic echocardiographic evaluation performed within the study period;
• clinical stability at the time of evaluation;
• ability to provide written informed consent.

Exclusion criteria

Patients were excluded if any of the following conditions were present:

• end-stage renal disease requiring chronic dialysis or prior kidney transplant tion;
• acute myocardial infarction or acute coronary syndrome within the preceding 3 months;
• severe heart failure (New York Heart Association class III–IV);
• active infection or systemic inflammatory condition at the time of evaluation;
• acute kidney injury at the time of H-FABP measurement;
• pregnancy or lactation;
• active liver failure or alanine aminotransferase (ALT) and/or aspartate aminotransferase (AST) levels >3 times the upper limit of normal;
• active malignancy or other severe systemic disease with limited life expectancy;
• missing essential variables required for the primary analysis.

This study was approved by the Ethical Committee of Grigore T. Popa University of Medicine and Pharmacy Iasi (no. 541/17 February 2025) and Dr. C.I. Parhon Hospital in Iasi, Romania (no. 4509/28 May 2024) and conducted in adherence to the international guidelines and the Declaration of Helsinki.

### 2.2. Measurements

For the present study, we evaluated general demographic variables (age, sex, height), comorbidities (hypertension, diabetes, etc.), parameters of CKD, such as serum creatinine, eGFR, and proteinuria/albuminuria, as well as ADPKD-specific characteristics (total kidney volume and Mayo classification).

#### 2.2.1. Echocardiographic Features

All patients underwent a comprehensive echocardiographic assessment performed using Philips CX50 Ultrasound System (Andover, MA, USA). The morphological parameters that were measured consisted of interventricular septum thickness, left ventricular posterior wall thickness, left ventricular dimensions (diameter and volumes), right ventricle dimensions (basal, mid- and apical diameters, area), left atrial dimensions (diameters, area, volume), right atrial dimensions (diameters, area), inferior vena cava diameter, left ventricular outflow tract diameter, ascending aorta diameter. The functional parameters we gathered included left ventricular ejection fraction (Simpson biplane), right ventricle function (tricuspid annular plane systolic excursion, TAPSE; tissue doppler S′ velocity; fractional area change, FAC), mitral pulsed wave doppler profile (E wave velocity, A wave velocity, E/A ratio, E wave deceleration time, pressure half time), lateral and septal tissue doppler velocities, aortic valve continuous doppler profile (maximum velocity, maximum pressure gradient), LVOT VTI, systolic index, cardiac index, tricuspid valve maximum velocity and gradient, pulmonary artery systolic pressure. All measurements and calculations were performed in accordance with the recommendations of the American Society of Echocardiography [19,20].

#### 2.2.2. Serum Levels of H-FABP

Peripheral blood samples collected in gel and clot activator vacutainer tubes were allowed to clot at room temperature and then centrifuged at 1000× *g*, and the resulting serum was divided into small aliquots and frozen at −80 °C until analysis. The H-FABP was analyzed by sandwich-enzyme linked immunosorbent assay method using commercially available assay (cat. no. E-EL-H1431, Elabscience^®^ Bionovation Inc., Houston, TX, USA). The analytical sensitivity of H-FABP was 0.09 ng/mL, and intra- and inter-assay coefficients of variation were 6.79 and 6.59%. The assay procedure, briefly presented here, includes the following: first all reagents and samples were brought to room temperature, then 100 µL of each dilution of standard, freshly prepared, blank and undiluted samples were added into appropriate wells, covered and incubated 90 min at 37 °C (on Matrix Orbital Delta plus incubator (IKA, Staufen, Germany); immediately after decanting the liquid, biotinylated detection Antibody working solution was added, followed by another hour of incubation; the plate was washed manually 3 times with previously prepared wash buffer and Horseradish Peroxidase (HRP) conjugate working solution was added and again incubated for 30 min at 37 °C; another step of washing followed the procedure (5 times) and a TMB (3,3′,5,5′-Tetramethylbenzidine) substrate reagent was added and incubated for 15 min at 37 °C; the stop solution terminated the enzyme-substrate reaction and the optical density of each well was determined at 450 nm using the BMG Labtech SPECTRO star Nano microplate reader, Germany and SPECTRO star Nano V6.20 software. The H-FABP serum concentrations were determined using the standard curve generated through MyAssays online v. R10.2 software (regression analysis with four-parameter logistic), by plotting the mean OD values and concentration of each standard.

### 2.3. Outcomes

#### Primary Outcome

The primary outcome was the association between circulating H-FABP levels and markers of subclinical cardiac involvement. The primary echocardiographic parameter of interest was the E/A ratio, selected a priori as a marker of transmitral filling dynamics. Since the assessment of diastolic function requires an integrated echocardiographic evaluation, tissue Doppler e′ velocities were also analyzed as complementary secondary echocardiographic parameters.

Secondary outcomes included:

• the relationship between circulating H-FABP levels and renal disease severity, defined by baseline kidney function (eGFR) and structural disease burden reflected by total kidney volume (TKV);
• the association between a composite H-FABP–renal severity score and echocardiographic parameters of diastolic function.

### 2.4. Statistical Analysis

All statistical analyses were performed using JASP. Continuous variables were expressed as mean ± standard deviation (SD) when normally distributed and as median (interquartile range) when appropriate. Categorical variables were presented as absolute numbers and percentages. Normality of distribution was assessed using the Shapiro–Wilk test and visual inspection of histograms and Q–Q plots. Circulating H-FABP levels were log-transformed before analysis due to skewed distribution. Univariable linear regression analyses were performed to assess the association between log-transformed H-FABP levels and baseline renal parameters (eGFR and total kidney volume), as well as echocardiographic indices of cardiac structure and diastolic function.

Multivariable linear regression models were constructed to evaluate whether the association between H-FABP and diastolic function parameters remained independent after adjustment for clinically relevant covariates, including age, sex, body surface area (BSA), and renal function (eGFR). Model assumptions, including linearity, homoscedasticity, normality of residuals, and absence of multicollinearity, were verified before interpretation of regression coefficients. Multicollinearity was assessed using variance inflation factors (VIF). Standardized β coefficients are reported to allow comparison of effect sizes across models. The proportion of explained variance was expressed using adjusted R^2^ values. All tests were two-sided, and a *p*-value < 0.05 was considered statistically significant.

A composite severity score was constructed to integrate renal function and myocardial injury. CKD stage and H-FABP levels were categorized into ordinal levels based on clinical classification and percentile distribution, respectively, and assigned 1 to 3 points. The individual components were summed, resulting in a total score ranging from 2 to 6.

Based on this composite score, patients were stratified into three categories: low risk (2–3 points), intermediate risk (4 points), and high risk (5–6 points). The severity classification was used as an ordinal variable in correlation analyses and as a grouping variable in non-parametric comparisons (Kruskal–Wallis test) of echocardiographic parameters.

## 3. Results

### 3.1. Baseline Characteristics

The cohort comprised 80 patients with ADPKD, with a mean age of 42.4 ± 10.4 years, and a predominance of female participants (61.3%). Mean estimated glomerular filtration rate was 70.8 ± 30.6 mL/min/1.73 m^2^, with serum creatinine levels of 1.24 ± 0.58 mg/dL, reflecting a wide range of renal function across the cohort.

With respect to disease severity, most patients were classified as Mayo imaging class 1C or 1D, while 17.5% were classified as class 1E. Hypertension was highly prevalent, affecting 63.7% of patients, whereas diabetes mellitus was uncommon (5.0%). Valvular disease was present in 23.8% of patients. The mean 24-h urine volume was 5.272 ± 2172 mL, consistent with the expected aquaretic effect of tolvaptan therapy.

Regarding treatment exposure, half of the cohort had been receiving tolvaptan therapy for more than 12 months, while 31.3% had initiated treatment within the previous 6 months. Concerning concomitant antihypertensive medication, 45.0% of patients were receiving ACE inhibitors or angiotensin receptor blockers, 27.5% calcium channel blockers, and 26.3% beta-blockers.

All baseline characteristics are presented in Table 1.

### 3.2. Primary Outcomes

The primary outcome assessed the association between circulating H-FABP levels and markers of subclinical cardiac involvement.

- Structural cardiac parameters

Circulating log-transformed H-FABP levels were significantly associated with markers of left ventricular structural remodeling. Higher H-FABP concentrations correlated with increased interventricular septal thickness (IVSd) (β = 0.078, *p* = 0.006) and greater left ventricular end-systolic volume (β = 0.009, *p* = 0.014).

Left ventricular mass, calculated using end-diastolic diameters in accordance with ASE/EACVI recommendations [21], showed a borderline association with H-FABP (*p* = 0.052). Left ventricular mass index could not be calculated due to the absence of anthropometric data in the study cohort. These findings are presented in Table 2.

B. Diastolic function parameters

More solid associations were observed with indices of diastolic function. Higher H-FABP levels were inversely associated with peak E velocity (β = −0.006, *p* = 0.023) and positively associated with peak A velocity (β = 0.010, *p* < 0.001). Consequently, a strong inverse association was found with the E/A ratio (β = −0.466, *p* < 0.001).

Similarly, H-FABP levels were inversely associated with tissue Doppler indices of myocardial relaxation, including lateral e’ (β = −0.039, *p* = 0.004), medial e’ (β = −0.059, *p* = 0.003), and averaged e’ (β = −0.057, *p* = 0.001). These findings are presented in Table 2.

C. Multivariable analysis

In multivariable linear regression analysis including eGFR, age, sex, and E/A ratio, both eGFR (B = −0.004, standardized β = −0.305, *p* = 0.046) and E/A ratio (B = −0.301, standardized β = −0.355, *p* = 0.020) remained independently associated with log-transformed H-FABP levels. The overall model was statistically significant (F = 6.165, *p* < 0.001) and explained 32.0% of the variance in circulating H-FABP concentrations (adjusted R^2^ = 0.320). These findings are in Table 3.

### 3.3. Secondary Outcomes

#### 3.3.1. H-FABP and Baseline Renal Disease Severity

The association between circulating H-FABP levels and baseline renal disease severity was assessed using eGFR and total kidney volume (TKV). Higher log-H-FABP concentrations were significantly associated with lower eGFR at baseline (β = −43.98, standardized β = −0.535, *p* < 0.001), with the model explaining 28.6% of the variability in eGFR (R^2^ = 0.286). In parallel, log-H-FABP levels were positively associated with total kidney volume at initiation (β = 1265 mL, standardized β = 0.299, *p* = 0.008), accounting for 9% of the variability in VRT (R^2^ = 0.090). These findings are presented in Table 2. These findings suggest that higher circulating H-FABP levels may be associated with impaired renal function and greater structural disease burden at baseline.

#### 3.3.2. Composite Severity Score and Echocardiographic Parameters

To further explore the potential combined relationship between renal dysfunction and myocardial injury, an exploratory composite severity score integrating CKD stage and H-FABP levels was constructed. The components of the scoring system are presented in Table 4.

The score was designed as an exploratory, hypothesis-generating approach based on CKD stages represented within the study cohort and H-FABP percentile distribution. The score was intentionally restricted to CKD stages 1–3, as no patients in the present cohort had CKD stage 4 or 5, consistent with the relatively preserved renal function observed across the study population (mean eGFR 70.8 ± 30.6 mL/min/1.73 m^2^). The H-FABP cut-offs were derived from the 25th and 75th percentile distribution within the cohort and should therefore be interpreted as population-specific thresholds rather than generalizable reference values. Spearman correlation analysis demonstrated that increasing severity risk class was significantly associated with multiple echocardiographic parameters, predominantly reflecting diastolic function. Higher risk class was associated with lower peak E velocity and higher peak A velocity, resulting in a significant reduction in the E/A ratio. In addition, significant negative correlations were observed with tissue Doppler e′ velocities, including lateral, medial, and averaged e′, indicating impaired myocardial relaxation. These correlations are summarized in Table 5.

Group comparisons further supported these findings. Significant differences across severity groups were observed for key markers of diastolic function, with the most pronounced differences between the low- and high-risk groups (Table 6).

**Figure 1:**
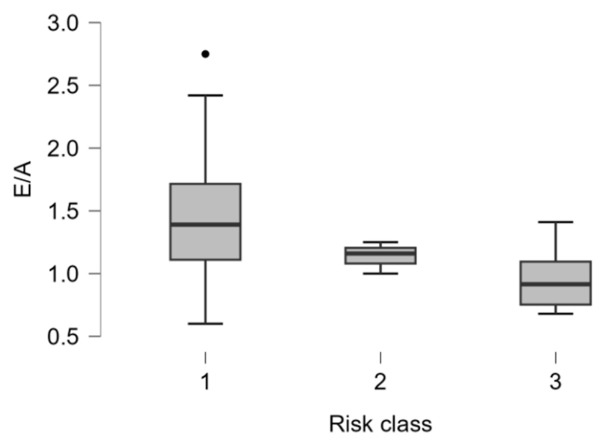
Boxplot of E/A Ratio Across Severity Risk Classes.

The E/A ratio progressively decreased with increasing severity, reflecting impaired early diastolic filling. This pattern is illustrated in Figure 1.

**Figure 2:**
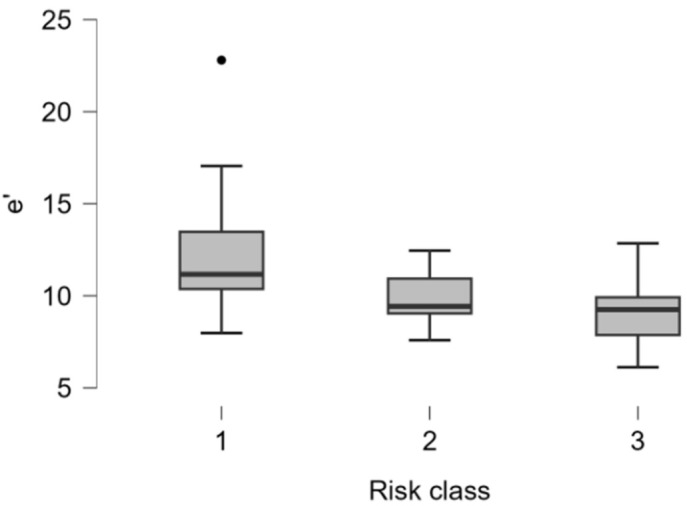
Boxplot of e′ Velocity Across Severity Risk Classes.

Similarly, tissue Doppler e′ velocities progressively decreased across increasing severity classes, indicating impaired myocardial relaxation. This pattern is illustrated in Figure 2.

Overall, these findings indicate a progressive impairment of diastolic function across increasing severity classes, characterized by reduced myocardial relaxation and increased reliance on atrial contraction, while no significant associations were observed with systolic function parameters.

## 4. Discussion

### 4.1. Our Findings

In this single-centre cohort of 80 patients with ADPKD treated with tolvaptan, circulating H-FABP levels were associated with both renal disease severity and markers of subclinical cardiac involvement. Higher log-transformed H-FABP concentrations were linked to lower baseline eGFR, greater total kidney volume, indicating an association with both functional and structural renal burden.

Beyond renal parameters, H-FABP showed significant associations with echocardiographic markers of cardiac remodelling, particularly echocardiographic indices suggestive of impaired myocardial relaxation, including the E/A ratio and tissue Doppler e′ velocities. Importantly, the association between H-FABP and E/A ratio remained significant after adjustment for age, sex, and renal function, suggesting that the observed connection was not fully attenuated after adjustment for renal clearance.

Furthermore, the integration of H-FABP with renal function into a composite severity score was associated with a graded trend across echocardiographic markers suggestive of impaired myocardial relaxation, with less favorable echocardiographic profiles observed across increasing risk categories.

Collectively, these findings suggest a potential role for H-FABP as a complementary biomarker that may reflect both renal disease burden and early subclinical myocardial alterations in ADPKD, though prospective confirmation is required.

### 4.2. H-FABP and Renal Disease Severity and Progression

Given that heart-type fatty acid–binding protein is primarily eliminated via renal filtration, circulating levels are expected to increase as kidney function declines, making renal clearance an important determinant of its systemic concentration. Supporting the concept that circulating H-FABP may reflect renal vulnerability, Oezkur et al. [17] showed that preoperative median H-FABP concentrations were significantly higher in patients who developed postoperative acute kidney injury (AKI) compared with those who did not (2.9 ng/mL vs. 1.7 ng/mL. In the cohort of 70 cardiac surgery patients, AKI occurred in 45 (64%), and preoperative H-FABP was also correlated with prolonged ICU stay, suggesting that higher circulating H-FABP identifies individuals at increased risk of renal injury under conditions of physiological stress [17]. H-FABP, also known as FABP-3, was evaluated in a much larger multicenter prospective cohort of 1219 high-risk patients undergoing cardiac surgery (TRIBE-AKI study), in which Schaub et al. [22] demonstrated that both preoperative and early postoperative plasma H-FABP levels were independently associated with AKI. First postoperative log (H-FABP) was strongly associated with severe AKI (adjusted OR 5.39; 95% CI 2.87–10.11 per unit increase), while preoperative levels were associated with any AKI (adjusted OR 2.07; 95% CI 1.48–2.89) and long-term mortality (adjusted HR 1.67; 95% CI 1.17–2.37). Notably, these associations persisted even after adjustment for change in serum creatinine and established biomarkers of renal and cardiac injury, suggesting that H-FABP may capture a distinct pathophysiological signal beyond reduced renal clearance alone [22].

Corroborating this, Shirakabe et al. demonstrated that among cardiac biomarkers measured at admission in patients with acute heart failure, s-H-FABP was the only independent predictor of true worsening renal failure in multivariate analysis (OR 5.472, 95% CI 2.729–10.972), further supporting the notion that H-FABP captures a distinct cardiorenal signal beyond passive renal retention [16].

In contrast to the acute perioperative setting, ADPKD represents a chronic and progressive model of kidney injury, characterized by sustained structural expansion and gradual functional decline. In ADPKD, disease progression is primarily assessed using established markers such as TKV [23], which reflects cystic growth and structural burden, and eGFR, which captures functional deterioration over time [24]. These parameters are central to risk stratification and therapeutic decision-making; however, they predominantly describe the renal component of the disease and do not directly account for the complex cardiorenal interactions that accompany disease progression.

In this context, our findings extend previous observations to a chronic cardiorenal setting. In patients with ADPKD, circulating H-FABP levels were closely associated with baseline renal function and structural disease burden. The consistency of these relationships supports the hypothesis that H-FABP may reflect ongoing cardiorenal stress in progressive kidney disease, rather than representing a passive consequence of reduced renal clearance.

### 4.3. H-FABP and Subclinical Cardiovascular Involvement

#### 4.3.1. Structural Cardiac Parameters

H-FABP was extensively evaluated across multiple cardiovascular conditions, in both acute and chronic settings. Clinical studies investigated its role as a biomarker in stable coronary artery disease, acute myocardial infarction [25], chronic and acutely decompensated heart failure [26], postoperative ventricular dysfunction (including after coronary artery bypass graft surgery), as well as in pulmonary embolism associated with right ventricular strain [27]. In these contexts, H-FABP has demonstrated utility both as an early marker of myocardial injury and as a prognostic indicator of adverse cardiovascular events, mortality, and recurrent hospitalizations. Importantly, beyond its renal associations, circulating H-FABP has also been shown to reflect ongoing subclinical myocardial injury in chronic cardiac conditions, particularly in heart failure, where elevated levels correlate with structural remodelling and an adverse cardiac phenotype [26].

This prognostic relevance was demonstrated also in patients with stable coronary heart disease, where elevated H-FABP levels were independently associated with a more than two-fold higher risk of composite cardiovascular events over 24 months (32.36% vs. 15.78%, *p* < 0.001), as well as cardiovascular death and acute heart failure–related hospitalization [28].

While most prior studies have focused on hard clinical outcomes, less attention has been paid to the relationship between circulating H-FABP and early structural cardiac alterations in chronic cardiorenal conditions. In our cohort of patients with ADPKD, higher H-FABP levels were significantly associated with markers of left ventricular structural remodelling, including increased interventricular septal thickness and a trend toward higher left ventricular mass. These findings may suggest that elevated H-FABP concentrations are associated with early structural alterations of the myocardium.

#### 4.3.2. Diastolic Function Parameters

Beyond structural remodelling, diastolic dysfunction represents an early and sensitive marker of myocardial impairment, often preceding overt systolic abnormalities. In chronic and acute heart failure, elevated H-FABP levels were consistently associated with ventricular dysfunction and adverse remodelling, reflecting ongoing cardiomyocyte injury and activation of inflammatory pathways, including the TNF and Fas/Fas ligand system, as demonstrated by Arimoto et al. [29], who showed that H-FABP serves as an independent marker of sustained myocardial damage and subsequent cardiac events in chronic heart failure.

Studies shown that H-FABP reflects more accurately ongoing myocardial injury than troponin T in chronic heart failure, with circulating levels correlating with disease severity and an increased risk of non-fatal cardiac events and mortality [26]. Importantly, higher baseline H-FABP concentrations were linked to parameters of left ventricular remodelling, and persistently elevated levels, even at discharge after acute decompensation, were associated with poorer outcomes [30]. Moreover, combined assessment of H-FABP with natriuretic peptides was shown to improve risk stratification in patients with ventricular dysfunction, suggesting that H-FABP captures a complementary pathophysiological dimension related to ongoing cardiomyocyte injury [31].

In line with these observations, our findings extend this pathophysiological framework to patients with ADPKD. In addition to the associations observed at the individual biomarker level, the composite severity score showed a graded trend across echocardiographic indices of diastolic function, with less favourable profiles observed across increasing risk categories.

This pattern supports the concept that combined renal dysfunction and subclinical myocardial injury contribute synergistically to early impairment of myocardial relaxation.

All patients in the present cohort were receiving tolvaptan at the time of evaluation. Boertien et al. demonstrated that short-term tolvaptan treatment significantly reduced urinary H-FABP excretion by approximately 57% in patients with ADPKD, with reductions in tubular injury markers observed independently of baseline kidney function [32]. The persistence of significant associations between circulating H-FABP levels and reduced eGFR, increased TKV, as well as echocardiographic indices suggestive of impaired myocardial relaxation despite ongoing tolvaptan therapy may indicate that these relationships are not solely attributable to passive biomarker accumulation. These findings provide additional support for the potential relevance of H-FABP as an integrative cardiorenal biomarker in ADPKD. Nevertheless, the cross-sectional design precludes assessment of the longitudinal impact of tolvaptan on circulating H-FABP or cardiac parameters, and prospective studies evaluating serial H-FABP measurements before and after tolvaptan initiation are warranted.

## 5. Clinical Implications

The present findings suggest that circulating H-FABP may provide complementary information to established markers of disease severity in ADPKD, such as TKV and eGFR. While these parameters remain central to risk stratification and therapeutic decision-making, they primarily reflect structural and functional renal involvement. In contrast, H-FABP may reflect the coexistence of renal disease burden and subclinical myocardial impairment, particularly echocardiographic indices suggestive of impaired myocardial relaxation, which may represent an early manifestation of cardiovascular involvement in ADPKD.

Furthermore, the composite severity score integrating H-FABP and renal function may provide an exploratory approach for integrating renal and cardiac parameters in ADPKD. However, its potential clinical applicability requires prospective validation in larger cohorts.

The independent association between H-FABP and diastolic parameters, even after adjustment for renal function, is consistent with the hypothesis that H-FABP may identify a more vulnerable cardiovascular phenotype in this population. If confirmed in larger prospective cohorts, H-FABP could contribute to a better understanding of cardiorenal interaction and may warrant further evaluation as a complementary cardiovascular biomarker in ADPKD.

## 6. Strengths and Limitations

This study has several strengths. First, it represents, to our knowledge, the first evaluation of circulating H-FABP levels in a well-characterized cohort of patients with ADPKD. Second, both renal and cardiac parameters were comprehensively assessed, allowing exploration of H-FABP within a true cardiorenal framework.

However, several limitations must be acknowledged. The study was conducted in a single center and included a relatively modest sample size, which may limit generalizability. In addition, several clinically relevant variables, including detailed cardiovascular history (atrial fibrillation, coronary artery disease, and prior heart failure), blood pressure control, smoking status, lipid profile, and albuminuria data, were not consistently available for all participants and may have contributed to residual confounding influencing the observed associations. The observational design also precludes causal inference. Although multivariable adjustments were performed, residual confounding cannot be excluded. In addition, given the exploratory nature of the echocardiographic analyses and the number of tested variables, the possibility of type I error related to multiple comparisons should also be considered. Furthermore, H-FABP levels are influenced by renal clearance, and despite adjustment for eGFR, partial retention effects cannot be entirely ruled out. Another limitation of our study is linked to the inherent flaws of echocardiography. Although patients with poor acoustic windows were excluded and measurements were performed using standardized protocols, our study did not include data validation by a second examiner. Finally, the absence of hard cardiovascular endpoints limits the ability to establish direct prognostic implications for clinical events.

## 7. Conclusions

In patients with ADPKD treated with tolvaptan, circulating H-FABP levels were associated with renal disease severity and echocardiographic markers of subclinical cardiac involvement, particularly parameters suggestive of diastolic dysfunction.

The integration of H-FABP with renal function into a composite severity approach may further support the concept of an early cardiorenal interplay in ADPKD. Although these findings should be interpreted within the limitations of a cross-sectional observational study, they suggest a potential role for H-FABP as a complementary marker in the integrated evaluation of ADPKD severity, warranting further prospective validation.

## Figures and Tables

**Table 1:** Clinical and renal characteristics of the ADPKD study cohort.

Clinical Parameters	n	Mean ± SD/n (%)	Min–Max
Age (years)	80	42.4 ± 10.4	18–62
eGFR (mL/min/1.73 m^2^)	80	70.8 ± 30.6	22–135.1
Serum creatinine (mg/dL)	80	1.24 ± 0.58	0.50–3.24
Sex	80		-
– Male		31 (38.8%)	
– Female		49 (61.3%)	
Mayo Imaging Class	80		-
– 1C		35 (43.8%)	
– 1D		30 (37.5%)	
– 1E		14 (17.5%)	
Hypertension	80	51 (63.7%)	-
Valvular disease	80	19 (23.8%)	
Diabetes mellitus	80	4 (5.0%)	-
Urine volume (mL/24 h)	79	5272 ± 2172	800–10,000
Antihypertensive treatment	80		
– ACEi/ARB		36 (45.0%)	
– Calcium channel blockers		22 (27.5%)	
– Thiazide diuretics		7 (8.8%)	
– Loop diuretics		1 (1.3%)	
– Beta-blockers		21 (26.3%)	
– Alpha-/central blockers		9 (11.3%)	
– SGLT2 inhibitors		0 (0%)	
Time from tolvaptan initiation	80		
– <6 months		25 (31.3%)	
– 6–12 months		15 (18.8%)	
– >12 months		40 (50.0%)	

**Table 2:** Linear regression analyses between log-transformed H-FABP, baseline ADPKD severity, renal function, structural echocardiographic and diastolic function parameters.

Dependent Variable	β (Unstandardized)	Standardized β	*p* Value	Adjusted R^2^
eGFR (baseline)	−43.98	−0.535	<0.001	0.277
VRT (initiation)	1265	0.299	0.008	0.078
IVSd	0.078	0.366	0.006	0.118
LVESV (4-chamber)	0.009	0.326	0.014	0.090
Left ventricular mass	-	-	0.052	-
Peak E velocity	−0.006	−0.303	0.023	0.075
Peak A velocity	0.010	0.469	<0.001	0.206
E/A ratio	−0.466	−0.550	<0.001	0.290
Lateral e′	−0.039	−0.387	0.004	0.134
Medial e′	−0.059	−0.393	0.003	0.138
Averaged e′	−0.057	−0.427	0.001	0.167

**Table 3:** Multivariable linear regression model for determinants of log-H-FABP.

Predictor	B (Unstandardised)	SE	Standardized β	t	*p* Value
Intercept	0.918	0.681	-	1.349	0.183
eGFR	−0.004	0.002	−0.305	−2.050	0.046
Age at initiation	0.001	0.005	0.030	0.205	0.839
Sex (0 = male, 1 = female)	−0.030	0.102	-	−0.292	0.772
E/A ratio	−0.301	0.125	−0.355	−2.401	0.020

**Table 4:** Composite Severity Scoring System Based on CKD Stage and H-FABP Level.

CKD Stage	Points	H-FABP Percentiles (ng/mL)	Points
1	1	≤1.576 (≤25th percentile)	**1**
2	2	1.577–4.413 (25th–75th percentile)	**2**
3	3	≥4.413 (≥75th percentile)	**3**

**Table 5:** Correlation Analysis Between Severity Risk Class and Echocardiographic Indices.

Relationship	Spearman’s ρ	*p*-Value
Severity risk class—BSA	0.270	0.044
Severity risk class—Peak E velocity	−0.342	0.010
Severity risk class—Peak A velocity	0.409	0.002
Severity risk class—E/A ratio	−0.572	<0.001
Severity risk class—Lateral e′	−0.503	<0.001
Severity risk class—Lateral a′	0.306	0.023
Severity risk class—Medial e′	−0.441	<0.001
Severity risk class—e′ (average)	−0.558	<0.001

**Table 6:** Differences in Echocardiographic Parameters Across Severity Risk Groups (Kruskal–Wallis Analysis).

Parameter	H Statistic	*p*-Value	Significant Post-Hoc Differences
E/A ratio	18.32	<0.001	Group 1 vs. 3 (*p* < 0.001)
e′ (average)	17.28	<0.001	Group 1 vs. 2 (*p* = 0.030), Group 1 vs. 3 (*p* < 0.001)
Lateral e′	13.68	0.001	Group 1 vs. 3 (*p* < 0.001)
Medial e′	11.82	0.003	Group 1 vs. 2 (*p* = 0.024), Group 1 vs. 3 (*p* = 0.003)
Peak A velocity	9.393	0.009	Group 1 vs. 3 (*p* = 0.007)
E deceleration slope	9.023	0.011	Group 2 vs. 3 (*p* = 0.018)
Peak E velocity	6.741	0.034	Group 1 vs. 3 (*p* = 0.045)
Lateral a′	6.184	0.045	Not significant post-hoc

## Data Availability

Data is available upon reasonable request from the corresponding authors.
